# Supplementary material for: MetaRibo-Seq measures translation in microbiomes
Source: Nat Commun. 2020 Jun 29;11:3268. doi: 10.1038/s41467-020-17081-z (PMC7324362; doi:10.1038/s41467-020-17081-z)
Supplement: Supplementary file 10 — Supplementary Data 7 [file 41467_2020_17081_MOESM10_ESM.zip › File2/Confidence_VeryHigh_Taxonomy/38064_out.krona.html]

Javascript must be enabled to view this page.

members
magnitude
magnitudeUnassigned
count
unassigned
taxon
rank

38064\_out

5

2
5
superkingdom

phylum
976
5

5
200643
class

order
171549
5

family
171550
1

1
239759
genus

species
214856
1

SRS019030\_contig\_number\_17770

815
2
family

816
2
genus

28116
1

SRS014459\_contig\_number\_contig-100\_5796.5797
species

species
162156
1

SRS053649\_contig\_number\_1786

family
2
171552

genus
838
2

species
2
1947174

SRS049896\_contig\_number\_27230SRS1041116\_contig\_number\_3839
